# Paradoxical reaction to IL-17A inhibitor: a case report and literature review

**DOI:** 10.3389/fmed.2024.1364127

**Published:** 2024-04-17

**Authors:** Jingyu Ren, Linjun Deng, Shuping Guo, Hongye Liu

**Affiliations:** Department of Dermatology, The First Hospital of Shanxi Medical University, Taiyuan, China

**Keywords:** psoriasis, pustular, IL-17 antagonist, adverse reactions, review

## Abstract

**Objective:**

A case of pustular psoriasis after treatment with secukinumab in a patient with plaque psoriasis is reported, which is the first case in China. To summarize the clinical characteristics of patients who developed the rare paradoxical reaction and treatment options received IL-17A antagonist therapy, we conducted a further literature review.

**Methods:**

Data were analyzed from a patient with plaque psoriasis who developed pustular psoriasis after treatment with secukinumab. A comprehensive review of relevant domestic and international literature was conducted, focusing on cases that met our inclusion criteria for analysis and synthesis.

**Results:**

Anti IL-17A therapy may lead to type conversion, with reported cases more prevalent in women and varying in onset time, predominantly involving palmoplantar pustulosis.

**Conclusion:**

Given the increasing use of IL-17A antagonists in psoriasis treatment, it is crucial to monitor for rare adverse reactions, including the paradoxical induction of pustular psoriasis.

## 1 Introduction

Psoriasis is one of the most common erythematous scaly diseases in dermatology. It is characterized by sharply defined patches with silvery scales on the surface accompanied by systemic manifestations (1). The etiopathogenesis is complex, and it is currently believed to be a chronic inflammatory disease mediated by the immune system against a genetic background (2). Recent studies have shown that IL-17A plays a critical role in the onset and progression of this disease (3, 4). IL-17A stimulates the excessive proliferation of keratinocytes and the production of related cytokines and chemokines, forming an inflammatory cycle (5). Anti IL-17A therapies have been shown to be very effective in plaque psoriasis and the main multi-center studies, SUPREME and its *post hoc* analysis, have demonstrated it (6, 7). It’s also important to note that paradoxial pustolosis has not been reported in the *post hoc* analysis of the SUPREME study with IL-17A inhibitors (6, 8). Herein, we report a case of pustular psoriasis in a 65 year-old male with plaque psoriasis after treatment with an IL-17A inhibitor. By reviewing relevant domestic and international literature, we summarize its clinical characteristics and treatment experience, thereby providing guidance for clinical practice.

## 2 Data and methods

### 2.1 Literature search and case inclusion criteria

Literature types included published case reports, clinical trails, observations, letters to the editor, editorials, commentaries, and conference papers. Exclusion criteria included patients with incomplete clinical data and irrelevant cases.

### 2.2 Search strategy

A literature review was performed to search for English and Chinese language articles from the construction of the database to 1 October 2023 using PubMed, Web of Science, Google Scholar, CNKI, Wanfang and Chinese Biomedical Literature databases. Search terms included “secukinumab,” “ixekizumab,” “bimekizumab,” “brodalumab,” “paradoxical,” “palmoplantar pustulosis,” “pustular,” and “psoriasis.” Additionally, manual retrieval and review of the literature were applied to discover all potential cases.

### 2.3 Study methods

A retrospective analysis of the data from 9 cases, including the current case, where IL-17A inhibitors were used to treat plaque psoriasis that then induced pustular psoriasis, was conducted. The characteristics of these cases were summarized.

## 3 Results

### 3.1 Case information

**Figure 1:**
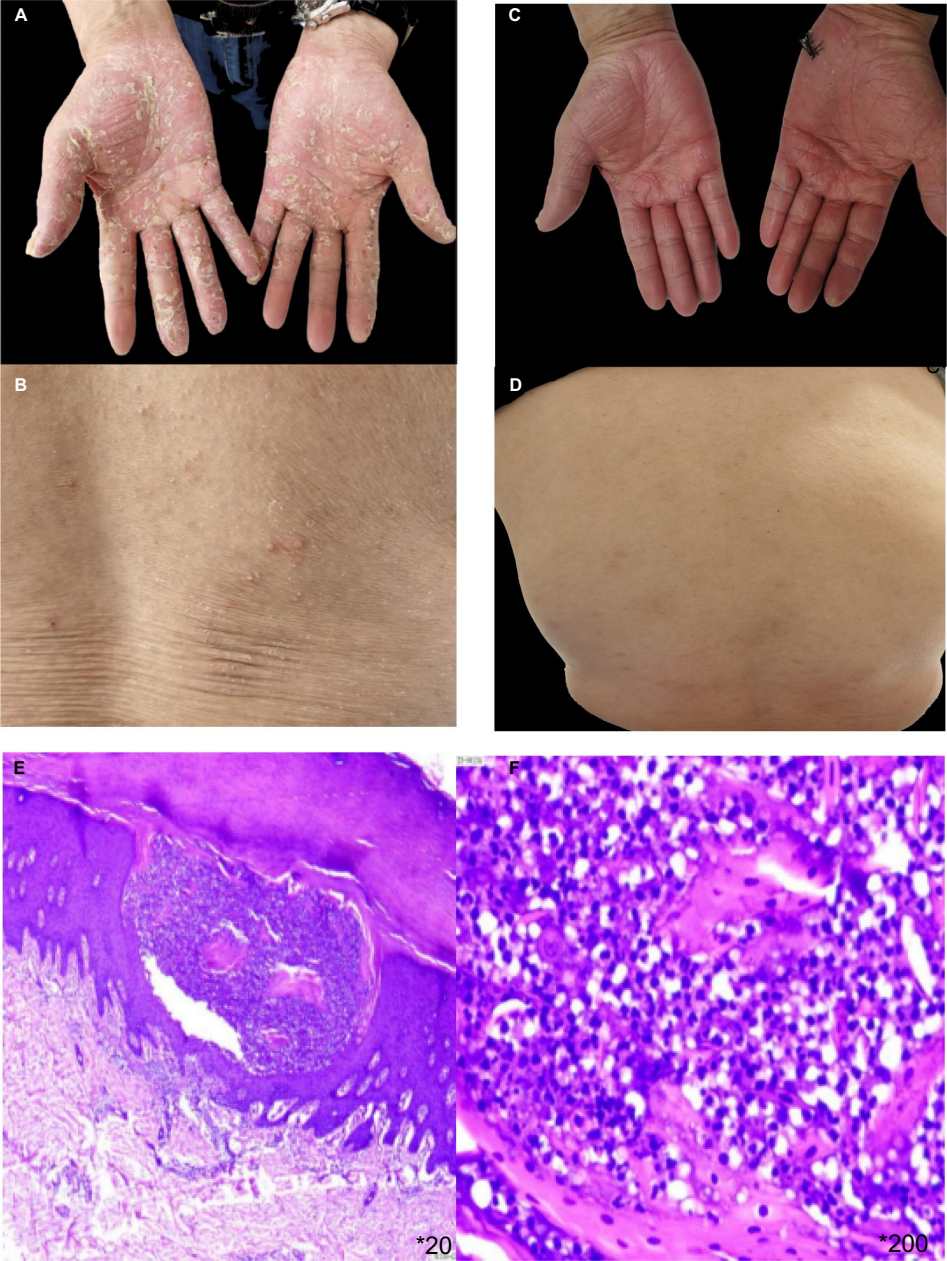
**(A,B)** erythema based pustules appeared on the trunk and palms of both hands after the 8th dose of secukinumab. **(C,D)** The pustulae of the trunk and palms of both hands completely subsided after local treatment and oral acitretin. **(E,F)** HE pathological staining showed epidermal psoriatic hyperplasia, neutrophil abscess in the upper granulosa layer, vascular dilation in the superficial dermis, and inflammatory cell infiltration dominated by peritubular lymphocytes.

The patient, a 65 year-old male, has been managing plaque psoriasis for 20 years, experiencing an exacerbation over last 10 days. Initially, erythematous scaly papules appeared on the back of his right hand without an identifiable trigger, accompanied by itching. It was not diagnosed or treated at the time, and the rash gradually increased, involving both elbows and both lower limbs. The presentation included multiple erythematous scaly patches, papules, and plaques of various sizes on both elbows and the extensor aspects of both lower legs, accompanied by itching. He was diagnosed with psoriasis at a local hospital and showed improvement after symptomatic treatment. Over the years, the patient’s skin lesions recurred and gradually worsened, appearing as erythematous scaly patches, papules, and patches of various sizes on the scalp, trunk, and limbs, accompanied by itching. He intermittently took acitretin, traditional medicine, and used topical treatments. The skin waxed and waned. Three months ago, after discontinuing topical treatments, dozens of erythematous scaly patches, plaques, and plaques reappeared on the patient’s trunk and limbs, accompanied by itching. He was diagnosed with plaque psoriasis at a local hospital and was treated with subcutaneous injections of secukinumab (by plaque psoriasis dosage form) for a total of 8 times. One month after starting the medication, the rashes on his trunk and limbs essentially subsided. After 3 months of treatment, dense pustules appeared on the erythematous bases of both his hands and feet, accompanied by a feeling of tightness, and scattered millet-sized pustules on the trunk (Figures 1A,B). He was subsequently admitted to our department. On examination: temperature was 36.5°C, pulse 75 beats/min, respiratory rate 18 breaths/min, and blood pressure 125/75 mmHg. No superficial lymphadenopathy was noted. No significant positive signs were found in the heart, lungs, and abdomen. No edema was seen in both lower limbs. There were scattered pustules on the trunk and lower limbs with surrounding erythema. Pustules the size of rice grains were symmetrically distributed on a reddish base on the palms and soles, some of which had dried up, with yellow scales visible. Culture and sensitivity tests of the pustules showed no bacteria growth. Histopathology of the palm pustules indicated a diagnosis consistent with pustular psoriasis (Figures 1E,F). This was thought to be an adverse reaction to secukinumab. After discontinuing secukinumab, the patient declined switching to another biologic agent such as TNF-α or IL-12/23 antagonist, he was treated with oral acitretin 20 mg/day, compound glycyrrhizin capsules 150 mg/day, and topical fluticasone propionate ointment twice daily. One month after treatment, The rashes on the patient’s trunk and limbs had mostly subsided (Figures 1C,D). He had no recurrence at a 6 month follow-up.

### 3.2 Literature review

#### 3.2.1 Literature search results

Based on our research strategy, we have retrieved 6 English language articles published from the construction of the database to 1 October 2023 in the database, covering a total of 8 patients. No relevant Chinese articles were found in the Chinese databases. Including our case, these findings bring the total number of cases to 9 for analysis (Table 1).

**Table 1:** Clinical data of patients.

Serial Number	Age	Gender	Original Disease	IL-17 Inhibitor Type	Induction Time	Pathology	New Pustular Psoriasis Type	Discontinued IL-17A Inhibitor	Treatment	Outcome	Association Evaluation
1[10]	22	Male	Severe Plaque Psoriasis	Secukinumab	32 weeks	Not done	Generalized Pustular Psoriasis	Yes	Switch to Infliximab +Cyclosporine	Complete Remission	Highly Likely
2[11]	61	Female	Severe Plaque Psoriasis	Secukinumab	12 weeks	Not done	Acrodermatitis continua	Yes	Switch to Ustekinumab+ topical methotrexate treatment	Complete Remission	Highly Likely
3[12]	50	Female	Severe Plaque Psoriasis	Ixekizumab	4 weeks	Consistent	Palmoplantar Pustulosis	Yes	Switch to Ustekinumab	Complete Remission	Highly likely
4[13]	26	Female	Plaque Psoriasis	Brodalumab	8 weeks	Not done	Palmoplantar Pustulosis	Yes	Topical Treatment + PUVA + Switch to Ustekinumab	Complete Remission	Highly Likely
5[13]	44	Female	Plaque Psoriasis	Secukinumab	64 weeks	Not done	Palmoplantar	Yes	Topical Treatment	Complete Remission	Highly Likely
6[13]	45	Female	Plaque Psoriasis	Secukinumab	14 weeks	Not done	Palmoplantar Pustulosis	Yes	Switch to Ustekinumab	Complete Remission	Highly Likely
7[14]	63	Female	Plaque Psoriasis with Psoriatic Arthritis	Secukinumab	36 weeks	Not done	Palmoplantar Pustulosis	No	Add Cyclosporine	Relief	Likely
8[15]	69	Female	Plaque Psoriasis	Brodalumab	16 weeks	Consistent	Palmoplantar Pustulosis	Yes	Switch to Cyclosporine	Complete Remission	Highly Likely
9	65	Male	Plaque Psoriasis	Secukinumab	12 weeks	Consistent	Palmoplantar Pustulosis and sporadic pustules on the trunk and limbs	Yes	Oral Acitretin, Compound Glycyrrhizin Topical fluticasone propionate cream	Complete Remission	Highly Likely

#### 3.2.2 Clinical features

Out of the 9 patients, 2 were male and 7 were female. The age of onset ranged from 22 to 69 years, with an average age of 49.44 years and a median age of 50 years. 8 cases of the primary disease were plaque psoriasis (9–13), and 1 case was psoriatic arthritis with plaque psoriasis (14). The time from the initiation of IL-17A inhibitors to the onset of pustular psoriasis, 1 case occurred at 1 month (11), 1 case at 2 months (12), 2 cases at 3 months (10), 2 cases at 4 months (12, 13), 2 cases at 9 months (9, 14), and 1 case at 16 months (13). The average time to onset was 5.6 months. Secukinumab, ixekizumab, and brodalumab were all implicated in inducing pustular psoriasis. Bimekizumab has not been implicated. The predominant type was palmoplantar pustulosis, with one case presenting as acrodermatitis-continua-like lesions and another as generalized pustular psoriasis (9–14). The IL-17A inhibitors used were secukinumab in 6 cases (9, 10, 12, 14), ixekizumab in 1 case (11), and brodalumab in 2 cases (12, 13). 3 patients underwent histopathological examination of skin tissue confirming pustular psoriasis (11, 13), and bacterial cultures were obtained from the pustules in three patients yielded negative results (9, 10, 12).

#### 3.2.3 Treatment and prognosis

The initial IL-17A inhibitor was discontinued in most cases. In one case, it was replaced with the TNF-α inhibitor infliximab, the condition completely resolved (9). In four cases, it was switched to the IL-12/23 inhibitor ustekinumab for treatment, the pustules completely disappeared (10–12). One patient continued the original biologic but was also treated with cyclosporine, resulting in effectiveness (14). Another patient stopped the original biologic and switched to the immunosuppressant cyclosporine, which was effective (13). One patient stopped the original biologic without undergoing further treatment, and still achieved improvement (12). In our case, the original biologic was discontinued and replaced with oral acitretin has also proven to be effective.

## 4 Discussion

Psoriasis is a chronic inflammatory disease mediated by immunity and controlled by multiple genes. Its chronic nature brings serious burden to patients’ life and psychological quality (15). The pathogenesis of psoriasis is complex and known pathogenic factors include TNF-α, IL-23, IL-17A, and so on (2). Currently, it is believed that T cells, especially TH17 cells and their related cytokines IL-17A, play a key role in its pathogenesis. A lot of clinical trails and real-world researches have demonstrated anti-IL-17A treatment has achieved remarkable effects in psoriasis (6, 7, 16). At present, the drugs of anti-IL-17A are mainly divided into three categories: (1) Anti-IL-17A monoclonal antibodies, such as secukinumab and ixekizumab (17, 18); (2) Anti-IL-17A/F monoclonal antibodies such as bimekizumab (19); (3) Anti-IL-17RA monoclonal antibodies, such as brodalumab (20). These drugs have been approved by the FDA for the treatment of psoriasis. Interestingly, case reports have indicated that different types of IL-17A inhibitors can induce pustular psoriasis (9–14), which is a well-known rare paradoxical reaction of anti TNF-α therapies (21). These cases were not accompanied by other causes of pustular psoriasis (such as infections, drug stimulation, etc.), and histopathology also confirmed the patients had developed pustular. This indicates that IL-17A inhibitors can, in rare cases, induce a transformation in the type of psoriasis.

The 9 patients in this study have the following clinical characteristics: (1) A higher prevalence in females, mainly among the middle-aged and elderly; (2) The use of secukinumab, ixekizumab, and brodalumab can all induce pustular psoriasis, there are no reports of paradoxical reactions induced by bimekizumab in the treatment of psoriasis, and we speculate that it may be related to the relatively short time on the market, and it needs to be continuously monitored for adverse reactions, especially in inducing such rare paradoxical reactions. (3) The timing of paradoxical reactions varies, from as short as 1 month to as long as 16 months; (4) The predominant newly developed psoriasis type is palmoplantar pustulosis; (5) Most patients with IL-17A inhibitor-induced pustular psoriasis exhibit mild clinical symptoms. By discontinuing the medication or switching to another monoclonal antibody or immunosuppressants, combined with local treatment, the condition can significantly improve or completely alleviate; (6) the clinical characteristics of skin lesions in most patients align with histopathological findings.

IL-17A inhibitors induce a phenotypic transformation in psoriasis, predominantly to palmoplantar pustulosis. The exact pathogenesis remains unclear. Potential mechanisms include: IL-17A inhibitors, by blocking IL-17A, may cause a cytokine pattern rearrangement in patients, leading to an increase in cytokines such as TNF-α (22). Overexpression of TNF-α results in excessive production of proinflammatory cytokines like IL-6, IL-8, IL-12, and IL-18, driving a transformation of the psoriasis phenotype to pustular psoriasis (23). On the other hand, IL-17A inhibition could induce a negative feedback loop in the IL-23/IL-17 axis, leading to elevated IL-23, which in turn stimulates TH-17 cells to produce other cytokines, such as IL-22. The latter can promote the proliferation and differentiation of keratinocytes. Activated keratinocytes can induce chemotaxis of neutrophils (IL-8), thus contributing to pustular psoriasis (24). Genetic factors may also be related to this adverse reaction, although definitive evidence is lacking (25). In summary, disrupting a particular cytokine network may lead to new autoimmune inflammatory phenomenon in patients treated with biologics, meriting further investigation to elucidate the causal relationship.

Regarding the management of patients who experience a phenotypic transformation of psoriasis while on IL-17A inhibitors, literature suggests that the core principle is to discontinue the IL-17A inhibitor. For mild cases, topical corticosteroids or phototherapy may be employed. For moderate to severe cases, the systemic administration of immunosuppressants, retinoids (such as acitretin), or switching to a different class of biologic, such as TNF-α antagonists or IL-23 antagonists, may be advisable. All patients experienced complete resolution after adjusting their treatment regimens (9–14). In our case, the patient’s condition gradually stabilized after discontinuing secukinumab and starting oral acitretin, which is recommended as the first-line treatment for pustular psoriasis (26), with no new rash emergence and no significant recurrence observed during follow-up.

In conclusion, this is the first reported case in China of a patient with plaque psoriasis developing symptoms of pustular psoriasis after treatment with the IL-17A antagonist secukinumab. Consequently, clinicians should be aware that IL-17A antagonists may trigger atypical psoriasiform rashes. Future real-world clinical research and studies on related mechanisms should be conducted, aiming to summarize the characteristics of cases where a type transformation might occur. This would provide a basis for early clinical recognition and ensure the precision and safety of biologic treatment.

## Data availability statement

The raw data supporting the conclusions of this article will be made available by the authors, without undue reservation.

## Ethics statement

The studies involving humans were approved by Ethics Committee of the First Hospital of Shanxi Medical University. The studies were conducted in accordance with the local legislation and institutional requirements. The human samples used in this study were acquired from primarily isolated as part of your previous study for which ethical approval was obtained. Written informed consent for participation was not required from the participants or the participants’ legal guardians/next of kin in accordance with the national legislation and institutional requirements. Written informed consent was obtained from the individual(s) for the publication of any potentially identifiable images or data included in this article.

## Author contributions

JR: Writing-original draft. LD: Methodology, Writing-review & editing. SG: Writing-review & editing, Supervision. HL: Writing-review & editing.
